# Feeding by numbers: an ethnographic study of how breastfeeding women understand their babies' weight charts

**DOI:** 10.1186/1746-4358-1-29

**Published:** 2006-12-22

**Authors:** Magda Sachs, Fiona Dykes, Bernie Carter

**Affiliations:** 1 Maternal & Infant Nutrition and Nurture Unit (MAINN), Faculty of Health, University of Central Lancashire, Preston, PR1 2HE, UK; 2 The Breastfeeding Network, Paisley, Renfrewshire, PA2 8YB, UK; 3 Families, Children and Life Course Group, Department of Nursing, University of Central Lancashire, Preston, PR1 2HE, UK

## Abstract

**Background:**

Weighing breastfed babies has been the subject of some controversy as the previous international growth chart was largely based on data from infants fed infant formula. The concern that professionals may be misled by the charts into suggesting to mothers that they supplement unnecessarily was a major impetus for the World Health Organization's investment in a new growth chart. Evidence of interpretation in practice has been scant.

**Methods:**

An ethnographic study was conducted in a town in the Northwest of England to investigate this issue. In the first phase, women and health visitors were observed in the well-child clinic during clinic sessions and breastfeeding group meetings. In the second phase, longitudinal interviews with 14 women were conducted. Each woman was interviewed up to three times in the first six months after the birth of her baby, with a total of 35 interviews.

**Results:**

Mothers and health visitors focussed on weight gain with frequent weighing and attention to even minor fluctuations of the plotted line being evident. Women felt it important to ensure their baby's weight followed a centile, and preferred for this to be the fiftieth centile. Interventions included giving infant formula and solids as well as changing what the mother ate and drank. Women also described how they worried about their baby's weight. Little effective support by health professionals with breastfeeding technique was observed.

**Conclusion:**

Babies were weighed more often than officially recommended, with weighing and plotting being at the core of each clinic visit. The plotted weight chart exerted a powerful influence on both women's and health visitors' understanding of the adequacy of breastfeeding. They appeared to rate the regular progression of weight gains along the chart centiles more highly than continued or exclusive breastfeeding. Thus weighing and visual charting of weight constituted a form of surveillance under the medical gaze, with mothers actively participating in self monitoring of their babies. Interventions, by mothers and health visitors, were targeted towards increasing weight gain rather than improving breastfeeding effectiveness. Improvements in training are needed for health visitors in weighing techniques, assessing growth patterns – particularly of breastfed babies – and in giving information to women, if the practice of routine weight monitoring is to support rather than undermine breastfeeding.

## Background

### Weight monitoring and breastfeeding

Monitoring infant weight is an integral part of baby care in most countries [1]. Health workers take regular weight measurements, plot them on a growth chart to make growth patterns visible in comparison with the reference population and discuss this with the mother or other care-giver. If there are concerns arising from the weight, any action taken in response should be agreed between the health worker and mother. Use of other growth monitoring indices (length/height and head circumference measurements, etc.), referral for additional investigations, and more intense weight monitoring during the intervention are recommended [2,3]. The aim is for early identification of potential threats to infant health through poor feeding or care practices and speedy adjustment to ameliorate these, or rapid identification of organic disease and appropriate treatment. Charts based on data collected in the USA, and largely from babies fed infant formula, were adopted for international use in the 1970s [4].

Breastfeeding is an unparalleled form of infant nutrition, with six months' exclusive breastfeeding and continued breastfeeding alongside appropriate complementary feeding until at least two years recommended by the World Health Organization (WHO) for all infants globally [5]. Studies conducted through the 1980s showed that the growth patterns of infants predominantly, fully or exclusively breastfed differed from the international reference. Breastfed infants' weight rises more steeply than the reference curve in the early weeks, and then appears to gently dip or 'falter' from approximately three months [6,7]. This discrepancy was felt to be large enough to be leading health professionals to advise mothers of breastfed babies who were healthy, feeding well and gaining appropriately to give supplements or to stop breastfeeding [8]. In response, WHO invested in the collection of prospective longitudinal data from babies in six countries to create new standards for infant weight, and other growth indicators [9]. Women whose babies were included were intending to breastfeed exclusively for at least four months (the international recommendation at the time the study was devised) and received extra support from trained breastfeeding advisors. They, their partners, and others in their household were non-smokers. The families enjoyed socioeconomic conditions favourable to growth. The charts thus represent a prescriptive approach showing how babies ought to grow [9-11]. During the course of the development of the charts, a need for retraining health professionals in their use and interpretation was identified and materials for this are, at the time of writing, forthcoming [1].

There has been little investigation of how health professionals interpret babies' weight charts in practice and how information is given to mothers [12]. There is similarly little investigation into how women understand the messages they are given and how they use these in their on-going feeding decisions. Official policies usually do not provide detailed guidance on when to supplement and it is unclear what sources health professionals use in making recommendations. Renfrew et al. assert that "there is insufficient research to guide decisions about which [breastfed] babies may genuinely need additional feeds" and what level of weight loss should "result in supplementation" [13] (p. 43). In the vacuum of evidence, practice may vary widely as to what individual practitioners suggest and what mothers do. Powers identifies a number of interventions in breastfeeding technique, which may improve infant weight gain [14,15]. There are few guidelines for practitioners on how to calculate the amount of supplement which will support growth with minimal negative impact on breastfeeding, or on how to evaluate when supplementation is no longer needed and how to support the transition back to full breastfeeding.

Behague conducted an ethnographic study of the impact of infant weight monitoring on breastfeeding women in Brazil [16]. Mothers in the low socio-economic setting of a shantytown, who had previously identified themselves as having 'weak milk', responded to weight monitoring positively. They valued the air of scientific authority conveyed by being able to refer to the chart and its interpretation. However, as they placed a strong emphasis on keeping infants' weights up, some of them gave supplements in order to prevent falls against the chart centiles. Thus, weighing, while valued, impacted negatively on breastfeeding [16]. Other literature alludes to the impact of weight monitoring, but this appears to be the only previous study to have investigated it in detail.

### Experience in the United Kingdom

In the United Kingdom (UK) a system of clinics and the profession of health visitors – nurses with extra training – were developed during the early decades of the twentieth century in response to concern about the welfare of infants in industrial towns; both were later extended to universal state provision [17,18]. Although health visitors advise on infant feeding, they do not generally receive adequate training on breastfeeding [19]. Once discharged from midwifery care, women are eligible to attend clinic and many attend regularly, often once a week or once a fortnight [20]. Every clinic visit is likely to include weighing the baby. In contrast, an authoritative manual for community practice, based on best available research and written by senior paediatricians, recommends a frequency of routine weighing at five or six times between the first health visitor weighing at around ten days and nine months [21].

Baby weights are plotted on a chart included in the parent-held child health record (PCHR) issued for each baby born in the UK [22]. This chart (the UK90) includes data from babies who were breastfed initially, as well as some who were not, and some for whom no feeding data were provided. Some of the data are longitudinal and some cross-sectional and all were initially collected for other purposes [23,24]. This chart is likely to represent optimum breastfed baby growth less well than the new WHO chart, but not to present as poor a fit as the previous international chart. UK paediatricians endorsed it as the best chart for UK use, but are currently considering the new WHO standards, although international charts have not hitherto been used [25].

In 2000, the government infant feeding survey showed that 69% of new mothers in the UK ever breastfed, while 28% of babies received any breast milk by the age of four months [20]. 'Insufficient milk' is the most common reason given for stopping breastfeeding between one week and four months [20] (p. 134). This category combines several issues including 'baby not putting on enough weight' (Hamlyn, personal communication, 2004). The 'insufficient milk syndrome' appears to involve complex, inter-related biological, physiological, cultural and iatrogenic components [26-29]. Its relation to recorded weight gain, for UK mothers, remains poorly investigated [30]. Several small qualitative UK studies have indicated that where mothers focus closely on weight gain or weight fluctuations this does undermine breastfeeding and result in earlier supplementation and/or cessation [29,31,32]. However, in general, weight monitoring is suggested as being a reassurance for parents [21].

The data presented here are from the doctoral study of the first author and aim to illuminate the way clinic interactions and weighing episodes shape breastfeeding women's understanding of plotted weight gain and its influence on their on-going baby feeding decisions.

## Methods

This study used an ethnographic approach that focuses on how individual action relates to community norms, through targeted participant observation of interactions in a setting of interest. The researcher may take part in interactions in the setting as well as observing them. Individuals encountered are asked about why they are doing what they do, giving an opportunity to compare what people do with how they explain their actual and intended actions. Observation and initial interactions can help identify questions that are meaningful for the people being studied and these may be explored in further interviews. Observational and interview data are both used to construct a picture of the context and relationships in which actions and explanations occur [33-36].

There were two phases of fieldwork. In phase one the first author attended 20 sessions of a child health clinic in a town in the Northwest of England between May and December 2001. The clinic was selected on pragmatic grounds, as the first author acted as sole researcher and conducted all observations and interviews and travel was self-funded. The clinic is in a town in the Northwest of England, in which the researcher had not worked as a volunteer, minimising risk of role confusion. Health visitors confirmed that this clinic had a higher rate of breastfeeding women than most others in the town. The breastfeeding initiation rate at the local hospital was 54% at the time of the study, compared to 61% reported for the North of England as a whole [20].

During observations the researcher sat in the small clinic room, which contained one scale. The health visitor staffing the clinic introduced the researcher to women as they entered. As well as observing interactions between health visitors and mothers (and, very rarely, fathers), short interviews were requested and conducted with nine breastfeeding mothers, and longer, private interviews with each of the four health visitors working in the clinic. The researcher also attended the breastfeeding support group run at the clinic, attending 14 sessions; participating in discussions and taking notes. Seventeen women were observed in group sessions. Although the intention was for first-time mothers only to be interviewed, there were relatively few breastfeeding women attending the clinic (despite this area having one of the highest rates of breastfeeding in the town), so mothers of second babies were also included. Data were collected from mothers who were offering any breastfeeding; as one health visitor remarked, "If you limit yourself to women who are exclusively breastfeeding you won't have anyone to interview". Demographic information was not collected on women in either phase of the study.

Data collected took the form of taped interviews, and field notes. These notes were written up to provide a full account as soon as practical, and taped interviews were transcribed by the researcher. As women observed or interviewed in the clinic might or might not be seen again, transcripts were not routinely checked with participants. In phase two, the researcher referred back to previous interviews, giving women the chance to correct or confirm her interpretations.

In a study with a single researcher, issues of bias need to be guarded against. Throughout the study the researcher kept a reflective diary. This allowed her to reflect on how the experience of attending the clinic or interviewing as well as what was said or observed impacted on her. Issues from this, as well as the emerging analysis of data, were shared with the other two authors, both experienced researchers.

Data from phase one were preliminarily analysed to inform interviews in phase two. In the second phase, beginning in November 2002, 14 women were recruited through an information sheet distributed by the health visitors in the clinic. Any woman who was willing to participate told a health visitor, who informed the researcher, who then contacted the woman. The aim was to recruit women who were breastfeeding and no other criteria were suggested to health visitors since it was not clear at the start what difference varying characteristics might make. In the end there was diversity in weighing frequency, length and exclusivity of breastfeeding, and also in socio-economic circumstances (as noted through observation during interviews in women's homes).

The aim was to conduct the first interview as soon as possible after the mother's first contact with the health visitor (at around 10 – 14 days after the birth). The intention was to arrange a second interview when the baby was around three months old and a third at six months. The first interview with some women was later, due to delays in establishing contact in the busy days after the birth of a baby. Telephone contact was maintained between interviews, so that if the mother stopped breastfeeding the next interview could be arranged soon after this happened. If a woman ceased to breastfeed at all by the second interview, this was the last one conducted. Interviews were arranged in women's homes at times convenient to them. The interviews were open-ended but a set of sample questions were used as prompts to ensure areas of interest were covered [see Additional file 1]. Altogether in phase two, 35 interviews of 40 to 90 minutes were conducted by September 2003; the tapes were transcribed by the first author.

Consent was sought and received from the Local Research Ethics Committee. Permission for changes in the study design, such as the inclusion of mothers of second babies, was sought and obtained. Health visitors distributed information about the study to breastfeeding mothers in the weeks before phase one clinic observations began to women who were breastfeeding. When women entered the clinic the health visitor asked if they were still breastfeeding. Those who were not breastfeeding were not approached by the researcher. Any interactions were observed, as the researcher remained in the clinic room throughout a session, but were not included in analysis.

Breastfeeding women signed a consent form before their first interview and before arranging each interview at phase two they were asked if they were still willing to be included. Two women initially agreed to take part in phase two but withdrew from the study before a meeting took place. None of the women who were interviewed initially declined subsequent interviews. Pseudonyms are used in place of women's actual names.

All data were analysed using a grounded theory approach, which, as described by Strauss and Corbin, is an approach that explicitly focuses on producing theory from data [37]. Grounded theory emphasises that data collection and analysis are not distinct phases, but that initial analysis of what is seen and heard in the early phases of observation shape the future course of the work in a reiterative process [37]. In phase one, utterances or short interactions written up were assigned initial 'open' codes or short descriptors. These codes were then used to re-analyse the data to deepen analysis and investigate the properties and dimensions of each code [37]. Phase two data were analysed using the same approach and new codes and themes emerged, as well as a deeper understanding of some of the areas identified in phase one. For example, comments about babies' plotted weight following the centile line led to exploration of the data for instances where weight gain in numbers of ounces was talked about which led to questions in phase two which were then analysed to explore how women's usage changed over time. Theorising about the issues arose from the data and was constantly re-examined in relation to the data. The exploration continued until saturation, when no new insights or themes emerged from continued analysis.

## Results and discussion

During the course of the study no breastfeeding mother was observed in clinic or was interviewed whose baby was currently experiencing 'faltering' weight gain. One mother in each phase reported that her baby had lost a large amount in the first days after birth. The mother in phase one responded by introducing supplements, the mother in phase two woke her baby more often for feeding. Both these episodes were reported in retrospect so there was no chance to observe the reactions as they were happening. Several mothers expressed concern about short-term periods when weight gain was not as good as they hoped for; none of these babies was referred and all reported that fluctuations were considerably less than the threshold for referral.

Most women came to clinic weekly or fortnightly. The younger the baby, the more frequent was attendance. In phase two, three of the fourteen mothers recruited did not attend the clinic frequently; they were all mothers of second babies. One moved to an area where, she reported, local health visitors did not provide baby clinic sessions. One had attended a lively clinic with an active social group in another part of the country with her first baby and found this clinic disappointing, and the third decided not to take her baby for frequent weighing before the birth as she had found this unhelpful with her first baby. These women provided useful comparisons with the women who attended clinic frequently.

In phase one, every visit to the clinic by a mother with a baby under six months of age was seen to involve weighing the baby. Several women were observed attending and having their baby weighed weekly for several months. On some occasions the baby gained well one week, little the next and well again on the third. Health visitors never suggested that too frequent weighing magnified the effects of variations in voiding patterns or daily feeding differences so that such close scrutiny was counter-productive where there was no identified problem to monitor.

When the clinic was busy, women waited their turn for weighing, and had a discussion with the health visitor afterwards. It was notable that, even when there was only one mother present, the health visitor would weigh and plot before asking the mother if she had any concerns or if she wished to discuss feeding. Once when a woman had left her PCHR at home the health visitor told her "I can't say if the weight is OK, because I can't plot it". Babies were weighed naked and the health visitor could observe them generally and also talk with the mother. However, the plotted growth curve appeared to be seen as the authoritative, definitive measure of infant well-being, by both mothers and professionals.

### Following the centile line

Most UK health authorities adopted the PCHR, which includes the growth chart, during the 1990s. Prior to this, a small card was used and weights in pounds and ounces were written on it. The possible effects of the change from a list of weights attained to a visual depiction of the baby's growth in the parent's record have not been previously remarked on. As one health visitor was prompted to reflect when asked about this change, "The pictorial evidence . . . is really significant to a mum".

In clinic observations it was striking to hear weight described in relation to the chart centiles, as personal experience of having babies in the 1980s had led to the expectation that weight would be described in terms of number of ounces. In fact this latter sort of comment was not absent. Linda reported: "He has put on eight or nine ounces a week", while Emma described her son: "He's fifteen pounds and two ounces and he's only four months". However the relation to the centile was often the measure presented to mothers, for example one health visitor said, "Look at the chart, she is doing fantastic – she is following along the line here." Another spoke directly to the baby, "Perfect text book, aren't you? We could use you to show what the curve is supposed to look like". Mothers then adopted this measure, "Oh, he's following his curve, that's good".

#### Weight recorded in numbers or plotted on centiles?

In all phase two interviews women were asked whether they found having the weight in pounds and ounces (imperial measurements, rather than kilograms, are still in everyday use in the UK) written in their PCHR or the plotted centile chart more helpful in understanding how their baby was doing. At the first interview most first-time mothers responded in the same vein as Paula, who said; "it's the weight; that line doesn't mean anything". Jayne however received fuller information at the first health visitor home visit:

"She pointed out that . . . he dropped off from his high birth weight and now he's back up to the same line as when he was born . . . so I would say probably seeing it plotted on the graph [is more meaningful]."

By the second interview (when the baby was three months old) Rosemary commented, "I now take some notice of the chart . . . somebody's explained it and I've read it properly". At the third interview most women tended to find the chart useful, now that, as Una noted, "There's more points", indicating that the fuller picture had more meaning. Of course, by six months, many feeding and care decisions had already been taken.

Mothers of second babies had had experience of learning about the centiles with their first child and many used this measure from the beginning with this baby. However two of them spoke of negative effects of their first experience:

"I'll look at the line . . .and that's purely because of Mark, I think because we were so concerned with trying to get him out of the blue zone [the shaded area between the second and 0.4th centile line on the boy's weight chart]." (Kelly)

Tessa had decided before this birth that she would not seek regular weighing:

"All that should matter is that there's some weight gain over the months, and the lines . . . can be rather off-putting . . . I think if it goes off the line to a lower line, it looks practically like your baby's ill because those red lines [on the girl's chart] are so clear . . . [there] is the pressure to keep up with this line. Which I didn't want to do . . . I wanted to be able to just look [at her] and see that she was OK."

Sarah, a mother who was herself a health visitor, whose daughter was growing along the ninth centile, told me how it felt as she approached each weighing, "When I put her on, I think, 'Oo, has she put on enough?"' Although she had professional experience of interpreting the plotted weight chart, she focussed on these weekly weights:

"You just want to be sure that you're plodding on as you should be. I don't know, because I'm a professional . . . whether you do become a bit more paranoid about weight, because you're trying to do everything in a pure way, 'You should be stuck on that line'."

#### Preferred centiles

There was a strong expectation that the baby's weight would closely follow one centile line, with positive feedback from health visitors when this happened. At the same time, all centiles were not equal; some were more desirable than others. One health visitor said:

"And as much as you say to a mum, 'Look it really doesn't matter, the whole chart is normal and it's watching that the baby finds a pattern and continues on that pattern that's more important than actually worrying about where they are on the graph', you will get some mums that will be concerned because their baby's on the nought-point-three centile, not on the seventy-fifth like the girl next door."

A number of women expressed particular satisfaction when their babies' weights followed the fiftieth centile. Alex said, "They say that what she's putting on, she's on the average, its ideal". Others were pleased that their babies were on high centiles. Women who mentioned that their babies were following centiles below the fiftieth tended to find this less satisfactory.

Paula, whose baby was first plotted on a lower centile, said that she felt better after she started supplemental infant formula feeds and the weight steadied:

"As long as he is following a line I think that's all right; he's not losing any and he's not gaining too much, just steadily . . . at the beginning when I was just purely breastfeeding, that's when it was just not really following any lines, then it started getting pretty normal really, on the middle line."

Paula's use of 'normal' indicates that the chart has not been well understood. However, this could be due to the sort of messages received in clinic. One health visitor said, as she plotted the weight, "I'll show you how he's doing on his chart. He's doing really well, fantastic. He's going on the fiftieth centile here, that's average, he has settled there".

The growth curves of a breastfed baby in the early weeks rise faster and higher than those of the standard charts, so this normal breastfed baby growth could appear 'too high'. Some women mentioned concern about obesity as one perceived positive aspect of being on the fiftieth centile. Zoë said, "I am pleased she is on the fiftieth, with me being heavier, I might be giving her milk that's on the fattier side and it could be a cause of concern". Kelly described how her baby's weight had fluctuated, most recently increasing, "But he's still below the middle-y line, so he's not overweight or anything". As half of all babies will be above the fiftieth centile at any one time, the assumption that this alone might be a sign of overweight is unfounded [38].

#### Using weight to judge breastfeeding

Women were concerned about the adequacy of the milk they were giving their baby. An increasing weight gave women confirmation that breastfeeding was 'working'. Wendy said, "You know they're putting on weight and they're actually getting what they're supposed to". Sarah echoed this, "When you're breastfeeding, you're not 100% convinced of how much is going in, are you? It **is **the only way to tell, you need that reassurance that you're doing all right".

A general picture was created in which plotted weight was understood to be the most important measure of baby well-being and breastfeeding was understood to be a means to the end of ensuring growth which conformed to the centiles, and could be sacrificed to ensure this growth. Even minor weight fluctuations could cause concern and various types of action on the part of women. Thus the practice of weighing, plotting of weight and visualisation of the growth curve appears to constitute a powerful form of surveillance that both health visitors and mothers are engaged in. Foucault [39] refers to the medical gaze upon patients as being both a requirement of staff and of those under the gaze. Those under the gaze then start to self monitor in an attempt to perform the 'correct' behaviours. In this case the women were policing their baby's weight as an indirect way of monitoring their own breastfeeding performance. In this way, as with other health related behaviours that have become institutional norms, everyone is caught up in the surveillance of infant and young child growth through the practice of weighing and plotting.

### Interventions to improve weight gain

Women reported a number of different ways they tried to increase the weight gain of their babies. Some were suggested by health visitors, others were undertaken without seeking professional advice. They included: offering infant formula; offering 'solids' (complementary foods); changing the mother's intake; and changing weighing frequency or practice. The data were examined for all mentions of interventions, including the expected mention of changes in breastfeeding technique.

#### Breastfeeding technique

Interventions which might influence the weight by ensuring that the baby received an increased quantity of breast milk or an increased proportion of fat in the milk during feeding, such as expressing milk to use as a supplement, increasing the frequency of breastfeeds or seeking skilled evaluation of the physical positioning of the baby at and attachment to the breast during feeding, were rarely mentioned. Although there is little research evidence to support these interventions, experienced clinicians recommend these as both preserving breastfeeding and improving weight gain in many cases [13-15].

During observations and interviews the researcher made a number of field note entries indicating that breastfeeding women were often observed holding the baby in awkward positions which might indicate poor attachment. This sort of observation, rather than the close scrutiny as part of a feeding assessment, is not definitive, but is suggestive. Health visitors were not observed offering practical help with positioning and attachment in the clinic or during breastfeeding group sessions. During interviews they were asked if they gave such help during home visits to women (which were never observed). One said she had not needed to do this in the year since she had been in practice. Another described a mother having problems:

"I came and discussed it with colleagues, and there's a possibility that maybe the baby's not latching on. Which personally, I don't feel is a problem, because this baby is sucking for between 15 and 20 minutes, so it's obviously getting something. I went through different strategies of positioning with her and just generally reassured her."

She is describing giving general information rather than observing and suggesting specific adjustments through an entire feeding episode.

#### Changing mother's intake

Some mentions were made of changing the mother's intake to influence weight gain. One health visitor detailed how she advised women about self care:

"If babies aren't gaining a lot of weight, and mums are anxious about it, but other than that they're settled, personally I find if you give them advice about resting and eating themselves and fluids . . ."

One mother who had been worried about her son's weight, reported:

"I cut extra cakes and biscuits out and my milk nearly went. It was like when he came on to me, there was nothing there . . . I just started having little things like full fat cheese and an extra snack . . . and it made the difference." (Kelly)

However, Renfrew et al. note that "There is no evidence that dietary modification or manipulating fluid intake is of therapeutic benefit" [13] (p. 66).

#### Infant formula

Women in this study mentioned many reasons for giving feeds of infant formula, such as hoping to reduce frequent feeding and the desire for a regular, predictable feeding pattern. Indeed, the longer interfeed intervals and sleeping patterns of formula-fed babies are culturally understood as normal, so parents expect these to be physiologically normal, turning to the bottle to achieve them. The general understanding that infant formula was fairly benign and an inevitable part of a baby's feeding was reflected in Sarah's remark that she gave a bottle "Just to see if she would [take it], really". Tessa, whose older daughter was breastfed for two years and who planned to (and did) breastfeed this baby exclusively for six months said "My neighbour has been asking Bryony (older daughter) 'Are you going to help mummy with the bottles?"' Thus, when Kelly was worried about her son's weight she talked about her plan to give her son infant formula if this weight trend continued, "We're going to move to it anyway, aren't we, so it's just a question of when really".

Health visitors told me that there was no local protocol for establishing when infant formula might be an appropriate intervention to suggest, and this appeared to be left up to the individual professional and be influenced by her confidence in her own ability to support breastfeeding. Several women reported that a health professional suggested giving infant formula to improve weight gain:

"I had such a struggle with it . . . I could not get her over eight pounds until I gave her a bottle at night. My health visitor suggested it, for my own peace of mind." (Hannah)

"In seven weeks he didn't gain one pound. They said at the clinic when I had him weighed that I could give him a bottle before bedtime . . . The doctor said I could do that if I was worried." (Suze)

Both these mothers represented the suggestion as being made to allay their fears, rather than because the health professional was concerned for the physical well-being of the baby. Health visitors sometimes voiced their perception that the impetus to give infant formula lay with family forces:

"And then their mothers and friends say 'Oh, just give it a bottle. That's what it needs, just give it a bottle' . . . and they're tired and upset and run-down and the baby's not putting weight on and is crying all the time. Give it a bottle, and then it will stick in its tummy and they'll all smile and say 'Isn't that wonderful?"'

The overall impression was that infant formula was regarded not only as fairly harmless, but as an inevitable stage in baby feeding and there appeared to be no one assuming responsibility for its use as an intervention to improve weight gain and monitoring the outcomes.

#### Complementary solid foods

At the time of the study, the recommendation in the UK was that 'solids' or complementary foods be introduced between four and six months of age. Women spoke of offering solids to increase the weight gain of their babies. Kelly, who was the mother in the study who had the most concern about her baby's weight said:

"Last time I was just so, 'I want to carry on breastfeeding', I mean I do want to carry on breastfeeding, but not at the expense of his weight gain . . . When I spoke to [health visitor] I was thinking maybe I ought to be trying a bottle, but she said to try a little bit of solids."

Sarah, who had been concerned at her daughter growing along the ninth centile said: "I needed to start her on solids at 16 weeks . . . I got to 16 weeks and I was happy to get to 16 weeks". She also noted that "When I started weaning, and whether that's because you're feeding them in two ways, you're not so vulnerable about [weight]". Sarah was herself a health visitor, so may have used her professional experience that offering solids affected weight gain. One of the clinic health visitors noted:

"It wouldn't be unusual for health visitors to say, 'Well you could start weaning and that may help'. We would view that to be more positive than introducing formula."

Many weaning foods are low-density and may provide fewer calories per gram than milk, so the potential to increase weight is likely to be limited.

#### Changing weighing practices

Another response when babies were not gaining as much as hoped for was changing aspects of weighing practice. Health visitors reported using more careful practice in response to disappointing weight gains; one noted "I did say, don't come to clinic, I'll weigh on the same set of scales, 'cos the scales can all differ". Another said:

"This mother had been upset because the baby only gained two ounces – it was OK on the chart, but she was upset . . . I even weighed the baby again at home – in case the weight had been due to the different scale."

This indicated awareness that standard practice could yield weights which were misleading, but this information was not regularly shared with women. As babies were reported to often be weighed on different scales, and at different times of day, improving accuracy of weighing might give a better record, but it is questionable whether this should only be when there is a concern about weight.

Some mothers mentioned that they might weigh more frequently, like Olivia who said: "if I do not think he is growing I would go every other week" (rather than monthly). Others agreed with Ulrike who said, "They say if it gets you worried with the weight, don't do it every week". One health visitor agreed with the latter approach, "I would usually say: 'I would suggest that I wouldn't rush to have the baby weighed too quickly, so giving yourself time, and time for getting things going again"'. Another health visitor commented, "It'll be interesting to see if the mum can stay away". She also noted:

"Once . . . they've lost weight, it is hard then to say 'that's fine, we'll see you in a month'. You want to say, 'Come back next week and see how you've got on'. Partly maybe, that's reassurance for us, I don't know, but mainly to reassure mum. Hopefully when you see her next week they've gained. Or not gained – that's very difficult."

Women's interventions, some of which were advised by health visitors, thus centred on increasing weight gain and did not either take into account possibilities of changing breastfeeding to improve weight gain, or give priority to preserving breastfeeding itself. Health visitors appeared to feel they had little to offer in terms of suggesting changes to breastfeeding which might improve weight gains. There was no sense that either women or professionals felt the value of the first six months of exclusive breastfeeding was something to consider in the balance against a hoped for increase of weight through introducing infant formula or complementary solids.

### Emotional Response

Weighing the baby is expected to provide reassurance that all is well and the baby is growing adequately. The result of frequent close scrutiny of the weight gain pattern led to concern at very minor fluctuations. Women worried about the weight, although poor weight gain is more properly understood as a sign of possible difficulty which invites investigation, not in itself a disorder [40].

Suze, whose son's weight had fallen to the ninth centile by eight weeks said:

"His length and head are nicely following the graph . . . but weight is a worry. His bones are growing, I worry that he will have weakened bones."

The health visitor replied, "He will find his natural pattern. Don't worry as long as there is no nose dive". A fuller explanation might have been helpful, as catch-down growth, crossing several major centiles, is common in the early weeks [41].

Some women spoke about worrying almost as if it were an intervention or activity they undertook when the weight was of concern. Bethany responded to slight fluctuations in weight, saying:

"She's not put on any weight this week. The week before she put on 10 ounces – I thought she'd do the same again. One week and if there is no gain, I'll worry . . . what's different this week from last week? Why hasn't she put any weight on?"

Bethany here planned in advance to worry! Ulrike suggested the source of worry is very personal, saying, "If she doesn't gain weight you think: is there something wrong with her or is there something wrong with me?"

A health visitor described the response of both women and professionals:

"If it hasn't put on a lot of weight, you're on that roller coaster, where they haven't put weight on and mum's concerned, and you're having to go back, and you're having to weigh it, because they want to know why it's not put weight on and what's going on. And they worry."

Such comments suggested that health visitors felt relatively powerless to interpret normal weight fluctuations and they, too, felt dependent on further frequent weighing.

## Conclusion

This study examines the actions and statements of breastfeeding women in one town in the Northwest of England. These data may not be generalisable to the whole of the UK, however findings resonate with those of other studies [28,29,32].

Health visitors' ethos is to work with mothers to resolve any questions or issues with regard to baby and young child care, but in this study they appeared to be locked into hierarchical relationships in which they enforced the norms of the chart. This was only part of a larger hierarchy as health visitors were accountable to the health authority, and were also constrained by policies set in accordance with the requirements of local paediatricians and the national programme of child health surveillance [21]. The growth chart was a reminder of the medical and scientific edifice behind the health visitor and her practice [42]. Ultimately, too, mothers and health visitors were in partnership to manage babies – who thus sat at the bottom of the hierarchy. The need of health visitors to maintain a watch on babies permeates the atmosphere in which baby weighing is conducted. The fostering of the mutuality of the mother-baby relationship, in which breastfeeding can flourish, sits uneasily with goals of surveillance and attention to prescriptive weight norms.

It appears that the understanding of infant weight gain may have shifted from increases in numbers of ounces to an expectation that weight will follow the shape of the centiles on the UK90 chart. This change is speculated to have happened with the inclusion of the chart in the PCHR; it was noted that women became inducted in this understanding over their first months of clinic attendance. Health visitors and women spoke about the plotted weight, revealing an expectation that growth would follow a particular centile, and that fluctuations from this were of concern. In addition, although the chart is based on normal babies, the fiftieth centile appeared to be understood as the most normal line for growth, rather than the statistical middle of a population. Babies growing along lower centiles, particularly those in the bottom quarter of the chart, caused concern. Babies growing along higher centiles were generally not of so much concern. This latter finding may reflect a traditional preference for chubby babies and may change as current social discussion of the problems of childhood obesity is incorporated in women's understandings.

By examining reactions when a plotted weight dipped below the previous centile, it appeared breastfeeding was measured against this standard. Infant formula was often used and regarded largely as a benign progression from breastfeeding. This highly processed foodstuff was understood as a reliable supplement which would ensure weight gain. Only a superficial understanding of principles of breastfeeding management was evident, with little use of alteration of physical technique or breastfeeding frequency to influence effectiveness. In addition to giving supplements of infant formula and solid foods to keep babies following the line, women engaged in worrying as an active response to demonstrate their attention to possible threats to the baby. Since the deviations reported in these cases were actually minor and well within normal fluctuations, such worry appeared largely unnecessary and potentially negative in undermining faith in continued exclusive breastfeeding. Attention to meeting weight gain goals reinforced the atmosphere of surveillance and the purpose of managing the baby rather than fostering mutuality and interaction between mothers and their infants. In order to support both physical breastfeeding success and satisfaction with breastfeeding this approach may need to be re-examined.

Whatever decision is taken about adopting the WHO chart for use in the UK, it is clear that the need identified by the WHO Multicentre Growth Reference team for improved training in interpreting plotted growth, using growth charts, and imparting information to parents is real. Fewer, better quality weighing episodes with more time for discussion may be desirable. Clinic sessions could be rearranged so that each visit does not necessarily include weighing, and the emphasis broadened from the physical outcomes of growth to wider aspects of well-being and breastfeeding effectiveness, as well as satisfaction with the experience. Clear information about the desirable frequency of routine weighing could be included in the parent held record. Protocols which suggest how to proceed when weight falters should be developed, which include breastfeeding interventions, indications for infant formula supplementation and guidance for transitioning back to full breastfeeding. At the same time, training for health visitors in supporting physical aspects of breastfeeding, facilitating social support, and the fostering of a professional culture which supports breastfeeding are needed. Perhaps health visitors might aspire to practice that assures women of the physical effectiveness of breastfeeding without suggesting that this is the only dimension of importance. A comprehensive implementation of such measures is required if the aim of using weight monitoring to support the basic health and nutrition of UK babies is to be met.

## Competing interests

The author(s) declare that they have no competing interests.

## Authors' contributions

MS contributed to the study design, applied for ethical approval, carried out the fieldwork, transcribed interview tapes, analysed the data, and wrote the paper.

FD contributed to the study design, assisted with application for ethical approval, and with drafting the paper.

BC contributed to the study design, assisted with application for ethical approval, university degree committee paperwork for the study, and with drafting the paper.

## Supplementary Material

Additional file 1 Question prompts used during interviews. This is the list of questions taken by the researcher to interviews during phase two of data collection. These were used to ensure areas of interest were covered in each interview. Click here for file
