# Supplementary material for: Feeding by numbers: an ethnographic study of how breastfeeding women understand their babies' weight charts
Source: Int Breastfeed J. 2006 Dec 22;1:29. doi: 10.1186/1746-4358-1-29 (PMC1779265; doi:10.1186/1746-4358-1-29)
Supplement: Additional file 1 — Question prompts used during interviews. This is the list of questions taken by the researcher to interviews during phase two of data collection. These were used to ensure areas of interest were covered in each interview. [file 1746-4358-1-29-S1.doc]

**Additional file**

**Question prompts used during interviews**

Questions used as prompts during interviews with health visitors in phase one

- Where do you think mums get their idea of how often they should bring the baby for weighing?
- What relative value do you give to the weight on the chart and on other indicators of child health?
- What interventions are proposed if weights deviate from ideal on chart?
- Is weighing the baby any more or less important for breastfed babies than for bottle fed babies?
- Women in this study talked about midwives helping with fixing. Does HV do this? When and where?

Questions used as prompts during phase two interviews with mothers

These questions were used as prompts during interviews in phase two. Before each interview they were modified in the light of answers from previous interviews with that mother and an individual sheet printed out for each subsequent interview. Often most questions came up in the course of the interview, the prompt sheet helped ensure that all issues were covered.

How are things going? What is the baby called? How old is (baby name) now?

Thinking back to the first visit from the health visitor. . .. When was that?

What happened at that visit?

Did she weigh the baby?

How did you feel about that?

Why was that?

Have you been to the clinic yet? [later: thinking back to the last visit to the clinic. . .]

If yes:

When was that?

What happened?

How did you feel?

What do you hope to get out of going to the clinic?

Do you look forward to it or not?

When the health visitor weighs (baby name) what are you hoping for? Why?

The health visitor writes down the weight and she also puts it on the chart. Do you find one of these more helpful in understanding how (baby name) is doing?

After (baby name) was weighed did you change anything about how you fed him?

What?

Why? Was it something you decided, or did the health visitor or someone else suggest that?

What happened after you changed things?

How often is (baby name) weighed?

How did you decide that was a good time to take (baby name) to be weighed?

If second baby: – Is this different than what you did with your first baby? Why is that?

How is breastfeeding going for the two of you?

As well as breastfeeding (baby name) are you giving anything else? Like, formula, water, colic remedies, etc. Why did you decide to give (baby name) that?

Are you expressing your own milk?

Have you been giving it to (baby name)?

When are you giving it? (eg regular basis, when go out)

How are you finding that breastfeeding is fitting with the other things you want or need to do?

Have you asked the health visitors for any help or information on breastfeeding?

How was that?

How did you feel about what they suggested?

Did you follow any of the advice they gave you?

Was it helpful?

How do you feel about needing advice about breastfeeding?

Do you feel that the health visitor(s) has supported you in your choices about feeding?

Have you had help or information about breastfeeding from anyone else?

Who?

How was that?

Have you had any questions about breastfeeding or problems that you haven’t been able to find someone to help with?

Has anyone else in your family or any of your friends breastfed?

Have people in the family/friends said anything about breastfeeding?

How do you feel about that?

Have they talked about the baby’s weight?

How has that made you feel?

Have they been interested in seeing the weight chart in the red book?

Have you been to the breastfeeding support group?

If yes: Why did you go?

How did you feel about the group?

Did you weigh your baby there? If yes, how was that compared to clinic?

If no: any reason why not?

Have you thought about how long you might continue breastfeeding?

Would you say that breastfeeding is important to you?

Would you say it is important to the baby?

Is there anything else about the experience of breastfeeding and weighing that I haven’t asked that you think is important or interesting?
